# Basaloid Follicular Hamartoma of the Eyelid: A Case Report and Literature Review about an Unusual Lesion in the Ocular Region

**DOI:** 10.3390/diagnostics12010140

**Published:** 2022-01-07

**Authors:** Nuno Jorge Lamas, Ana Patrícia Rodrigues, Maria Araújo, José Ramón Vizcaíno, André Coelho

**Affiliations:** 1 Anatomic Pathology Service, Pathology Department, Centro Hospitalar e Universitário do Porto, Largo Professor Abel Salazar, 4099-001 Porto, Portugal; anapatriciarodrigues.10@gmail.com (A.P.R.); joseramonvizcaino@chporto.min-saude.pt (J.R.V.); andreclementecoelho@gmail.com (A.C.); 2 Life and Health Sciences Research Institute (ICVS), School of Medicine, University of Minho, Campus de Gualtar, 4710-057 Braga, Portugal; 3 ICVS/3B’s, PT Government Associate Laboratory, University of Minho, 4710-057 Braga, Portugal; 4 Ophthamology Department, Centro Hospitalar e Universitário do Porto, Largo Professor Abel Salazar, 4099-001 Porto, Portugal; u04747@chporto.min-saude.pt

**Keywords:** basaloid follicular hamartoma, eyelid, eyelid tumour, ocular pathology, ocular region

## Abstract

Basaloid follicular hamartoma (BFH) is a normally benign, uncommon, malformative lesion involving the hair follicles, which usually poses challenges in the differential diagnosis with other benign and malignant tumours, especially basal cell carcinoma, due to significant clinical and morphological overlap. Here, we report the case of a 53-year-old male who presented with a mass in the upper left eyelid evolving for one year. The patient had a previous history of total colectomy and an abdominal desmoid tumour within the context of Familial Adenomatous Polyposis (FAP), with a documented germline mutation in the Adenomatous Polyposis Coli (APC) gene. The eyelid lesion was biopsied and the histological analysis of the three small tissue fragments received revealed fragments with cutaneous–conjunctival lining displaying a subepithelial proliferation of basaloid nests with peripheral palisading, compatible with primitive hair follicles. There were images of anastomosis between different basaloid nests, which had their connection to the epithelial lining preserved. The stroma had high cellularity and sometimes primitive mesenchymal papillae were evident. Pleomorphism was absent, mitotic figures were barely identified, and no necrosis was seen. The basaloid nests did not have epithelial–stromal retraction nor mucin deposits. A diagnosis of BFH was proposed, which was later confirmed after surgical excision of the whole eyelid lesion. No evidence of carcinoma was present. This case illustrates the main features of the rare benign eyelid BFH. The standard medical or surgical approach of these lesions remains to be firmly established. Nearly nine months after surgical excision our patient remains well without signs of disease recurrence.

## 1. Introduction

Basaloid follicular hamartoma (BFH) is a rare benign lesion mainly consisting of a superficial malformation centred in the hair follicles [1]. The lesion was originally described by Brown and colleagues, in 1969, when they reported the case of a 32 year-old female patient with myasthenia gravis, abnormal urinary amino acid excretion and progressive generalized alopecia, which was shown to be related with the presence of a hamartoma of basaloid cells of each hair follicle [2]. Later, in 1985, Mehregan and Baker proposed the term BFH when describing three novel cases with similar clinical and histological features [3]. Clinically, BFH can present as a small individual skin-coloured to brown papule, or as multiple lesions located mainly in the face, scalp or trunk [1]. Interestingly, only three cases of BFH were previously reported in the eyelid [4,5,6]. Histologically, BFH is characterized by an epithelial proliferation of basaloid cells which can mimic benign hair follicle-based lesions or basal cell carcinoma (BCC), among other entities [1]. BFH can be a sporadic, congenital or familial condition, with some cases being associated with inherited genetic syndromes, namely mutations in the Patched-1 (PTCH1) gene on chromosome band 9q23 [1]. PTCH1 is a member of the same pathway also implicated in the nevoid BCC syndrome/Gorlin-Goltz syndrome [1,7,8]. Previous studies have also demonstrated an important association of BFH with systemic conditions such as alopecia [9,10], cystic fibrosis [11], myasthenia gravis [2,12] and systemic lupus erythematosus [9,10].

## 2. Case Presentation

**Figure 1:**
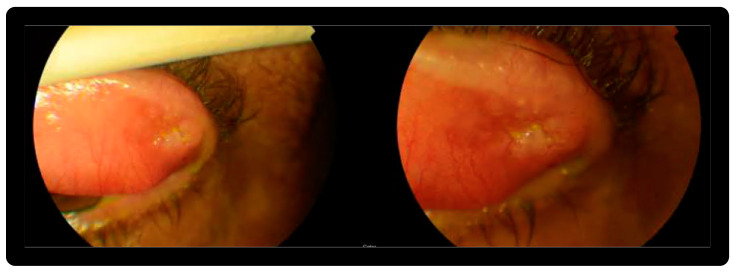
A 53-year-old man with a slightly elevated and palpable nodule measuring nearly 10 mm located in the lateral third of the upper left eyelid. A mild conjunctival hyperaemia was associated with the lesion, which had been progressively growing for nearly 1 year.

We report the case of a 53-year-old male who presented with a palpable 10 mm nodule in the upper left eyelid evolving for nearly one year, centred in the cutaneous-conjunctival transition area (Figure 1). The patient had a rich previous medical history, being submitted for total colectomy followed by ileorectostomy within the context of Familial Adenomatous Polyposis (FAP) when he was 30 years old. In the surgically removed colon, more than 100 adenomatous polyps were identified. An Adenomatous Polyposis Coli (APC) gene germline mutation was found in codon 232, exon 6. Several members of the family also carry mutations in the APC gene and have developed desmoid tumours, as well as, gastrointestinal (GI) tract polyps and tumours. Later, when the patient was 35 years old he presented with severe lower GI tract bleeding and an abdominal desmoid tumour was found during the episode. He was submitted to partial resection of the tumour and partial small intestine resection. After surgery, he was initially treated with sulindac and tamoxifen. Then, he underwent treatment with dacarbazine and doxorubicin for several months until he started daily meloxicam treatment. Six years ago he developed acute renal failure and meloxicam was suspended. Since then, he is under an intensive surveillance scheme and the abdominal desmoid tumour size and features have remained stable based on the findings of successive imaging studies performed. Over the past years, he has undergone routine GI tract surveillance endoscopies, which have led to the identification of several polyps in the rectum and ileo-rectal anastomosis area with adenomatous transformation harbouring low and high-grade dysplasia. Five years ago he developed a lesion in the lateral upper left eyelid, which was clinically considered a chalazion. This lesion was refractive to medical treatment and, thus, was surgically excised. No information regarding the pathological analysis of this initial eyelid lesion is available in his medical record. Two years ago, the patient noted a palpable nodular lesion in the same area of the lateral third of the upper left eyelid (Figure 1). The lesion was centred in the cutaneous–conjunctival transition area and had been growing slowly for nearly one year (Figure 1). Except for some conjunctival hyperaemia, there were no other local significant changes in the ocular region.

**Figure 2:**
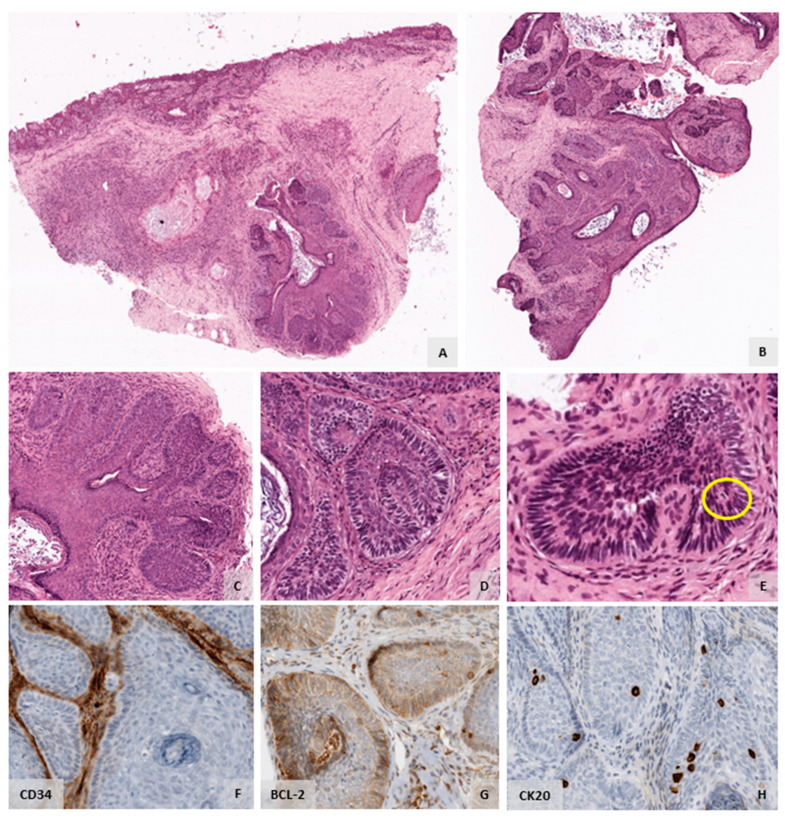
Basaloid Follicular Hamartoma (BFH) of the upper left eyelid (biopsy material). (**A**,**B**). The histological analysis of the 3 biopsy fragments received showed fragments with cutaneous–conjunctival lining displaying a subepithelial proliferation of basaloid nests (H&E, 20× magnification). (**C**). Peripheral palisading compatible with primitive hair follicles was evident. There was preservation of connection of the basaloid nests to the epithelial lining and images of anastomosis between different basaloid nests. (H&E, 100× magnification). (**D**). The tumour stroma had high cellularity and sometimes primitive mesenchymal papillae were evident. Most of the basaloid nests did not present epithelial–stromal retraction nor mucin deposition (H&E, 250× magnification). (**E**). Mitotic figures were scarcely identified (yellow circle). Pleomorphism and necrosis were absent (H&E, 250× magnification). (**F**). The immunohistochemistry study with antibodies for CD34 showed expression in the stromal cells surrounding the tumour and in blood vessels (250× magnification). (**G**). BCL-2 expression was limited to a few basal cells in the outermost portion of the lesion (250× magnification). (**H**). CK20 expression was observed in a few cells scattered within the tumour (250× magnification).

**Figure 3:**
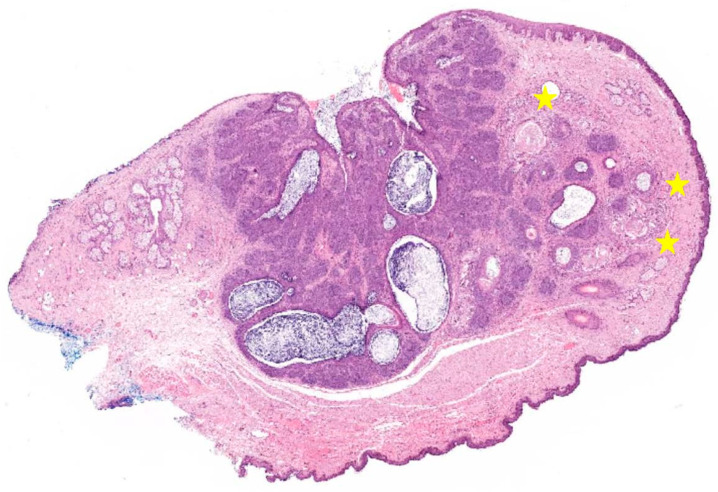
Basaloid Follicular Hamartoma (BFH) of the upper left eyelid (whole eyelid lesion). In the surgically excised, whole-eyelid lesion, one observed a basaloid malformative lesion centred in the cutaneous–conjunctival transition zone with features analogous to what had been previously described in the biopsy material (Figure 2). A mild foreign body type giant cell reaction was observed in the periphery of the tumour (yellow stars), most likely being associated with previous biopsy procedures. There was no evidence of malignant features and the diagnosis of BFH was confirmed (H&E, 4× magnification).

The eyelid lesion was biopsied by the ophthalmology team, and we received three elastic and whitish tissue fragments, measuring between 4 mm and 5 mm for those of the largest size. The histological analysis revealed fragments with cutaneous–conjunctival lining displaying a subepithelial proliferation of basaloid nests, with peripheral palisading, compatible with primitive hair follicles (Figure 2A,B). There was preservation of their connection to the epithelial lining and images of anastomosis between different basaloid nests (Figure 2C). The tumour stroma had high cellularity and sometimes primitive mesenchymal papillae were evident (Figure 2D,E). The cellular pleomorphism was absent, the mitotic figures were scarcely identified and necrosis was not identified (Figure 2E). The majority of the basaloid nests did not present any epithelial–stromal retraction nor mucin deposits (Figure 2E,F). The immunohistochemistry study, conducted using antibodies for CD34, showed expression in the stromal cells adjacent to the basaloid component and in blood vessels (Figure 2F). BCL-2 expression was only weakly and focally detected in the outermost basal cells (Figure 2G). CK20 expression was detected in a few cells within the lesion (Figure 2H). The diagnosis of BFH was proposed, which was later confirmed upon surgical excision of the whole eyelid lesion (Figure 3). Of note, in the excision specimen, there was only a slight foreign body type giant cell reaction in the periphery of the lesion, most likely related to the previous biopsy procedures (Figure 3). There was no evidence of carcinoma (Figure 3).

## 3. Discussion

BFH is a rare benign malformative lesion involving the hair follicles, normally limited to the superficial dermis [1]. There are only three previous cases of BFH reported in the eyelid [4,5,6]. The first case was published in 2012, concerning an 86 year-old female patient that had an asymptomatic lesion in the left lateral lower eyelid evolving for approximately 2 years [4]. Afterwards, there was a report about a 52 year-old male patient, who presented with a 10 mm solitary, asymptomatic, hyperpigmented, slow-growing left upper eyelid tumour, increasing for 4 years [5]. The most recently reported case is relative to a paediatric diabetic female patient of 6 years old, who presented with a flesh coloured papule in the upper eyelid evolving for approximately two years [6]. Therefore, our case represents the fourth published case of eyelid BFH, illustrating the main features of this unusual benign entity, which is rarely described in the eyelid.

BFH normally poses challenges in the differential diagnosis with other benign tumours of the follicular infundibulum (for example, trichoepithelioma) and malignant epithelial tumours, namely BCC, especially the infundibulocystic variant, due to significant morphologic overlap [1,13]. In our case, based on the morphology of the biopsy material, the main differential diagnosis was BCC, which was deemed to be less likely given that peripheral palisading was restricted to areas compatible with primitive hair follicles, epithelial–stromal retraction was not prominent, mucin deposits were absent, cellular pleomorphism was virtually non-existent, mitotic figures were scarce, and necrosis was not identified [1,13]. In addition, unlike in BCC, the immunohistochemistry study showed CD34 expression in the stromal cells around the basaloid nests and BCL-2 expression in the outermost basal cells, with only a few scattered CK20-positive cells within the lesion [1,13]. Another diagnostic possibility that was considered was trichoepithelioma, however, those lesions have a more pronounced nodular growth pattern and more conspicuous formation of keratin cysts, which was not evident in our case [1]. In addition, sebaceous carcinoma was also excluded, given the non-malignant morphological features of the lesion and the absence of signs of sebaceous differentiation [13]. Based on the lesion location, another theoretical, albeit even more remote diagnostic possibility to consider was Merkel cell carcinoma, which could be ruled out based on the bland morphological aspects of the lesion and the absence of CK20 generalized immunoreactivity [14,15].

Our patient is a member of a FAP family. Interestingly, FAP patients carrying a germline APC gene mutation might develop several skin and soft tissue benign lesions, such as epidermal cysts, fibromas, desmoid fibromatosis, pilomatricomas, and lipomas [16,17]. However, there are no previous studies in the literature reporting the development of BFH lesions in FAP patients. Further studies are needed to elucidate this possible association. Interestingly, in the ocular region APC gene mutations have been associated with pigmented ocular fundus lesions [18,19]. Remarkably, previous genetic studies found an association between BFH and PTCH1 gene mutations, which is a gene that normally encodes the patched-1 protein that acts as the receptor for the Sonic Hedgehog (SHH) protein [1,20,21]. The SHH pathway is fundamental in numerous aspects of embryonic development [20,22,23]. Dysregulation of the SHH signalling might lead to increased cellular proliferation and triggering tumour formation [21,23,24].

Even though BFH is typically considered a benign lesion, previous reports suggest that different forms of BCC could arise within the BFH context [25], which has led some authors to recommend the surgical excision of the whole lesions in cases of suspected BFH [26]. Nevertheless, the standard medical or surgical approach of BFH remains to be firmly established [1]. Of note, the identification of follicular bulbs, papillary mesenchymal bodies, trichohyalin granules, hair shafts, shadow cells, and focal CD34 staining in adjacent tumour stromal cells tends to be associated with benignancy [1]. In the cases where there is an associated autoimmune disease, the treatment of the underlying condition may lead to BFH regression [1].

Nearly 9 months after surgical excision of the lesion, our patient remains well, without any signs of disease recurrence. The patient is being kept under an intensive follow-up plan for his other underlying medical conditions.

## 4. Conclusions

We report here a solitary BFH arising in the lateral third of the upper left eyelid of a middle-aged male patient, who is a member of a FAP family. Reports of this typically benign basaloid tumour in the ocular region are scarce and, thus, it is important to document such cases to increase the awareness of the medical community to this disorder. This case illustrates the main features of this rare, non-malignant eyelid entity, which can display features analogous to other benign and malignant epithelial lesions, namely BCC, posing challenges to the accurate diagnosis. The potential of identifying malignant features within BFH has led to the precautionary recommendation of surgical excision in several cases. However, the gold standard medical or surgical approach of BFH remains elusive. A better understanding of the pathogenesis and natural evolution of BFH will help to establish in the future the optimal management approaches for these cases.

## Data Availability

Not applicable.
